# Genetic Determinants of Virulence in Pathogenic Lineage 2 West Nile Virus Strains

**DOI:** 10.3201/eid1402.070457

**Published:** 2008-02

**Authors:** Elizabeth M. Botha, Wanda Markotter, Mariaan Wolfaardt, Janusz T. Paweska, Robert Swanepoel, Gustavio Palacios, Louis H. Nel, Marietjie Venter

**Affiliations:** * University of Pretoria, Pretoria, South Africa; † National Institute for Communicable Diseases, Sandringham, South Africa; ‡ Columbia University, New York, New York, USA

**Keywords:** West Nile virus, flavivirus, phylogenetic analysis, genome sequence analysis, strain virulence, neuroinvasive, lineage 2, research

## Abstract

We determined complete genome sequences of lineage 2 West Nile virus (WNV) strains isolated from patients in South Africa who had mild or severe WNV infections. These strains had previously been shown to produce either highly or less neuroinvasive infection and induced genes similar to corresponding highly or less neuroinvasive lineage 1 strains in mice. Phylogenetic and amino acid comparison of highly and less neuroinvasive lineage 2 strains demonstrated that the nonstructural genes, especially the nonstructural protein 5 gene, were most variable. All South African lineage 2 strains possessed the envelope-protein glycosylation site previously postulated to be associated with virulence. Major deletions existed in the 3′ noncoding region of 2 lineage 2 strains previously shown to be either less or not neuroinvasive relative to the highly neuroinvasive strains sequenced in this study.

## Abstract 2

The most likely determinants are mutations in the nonstructural proteins encoding viral replication and protein cleavage mechanisms.

West Nile virus (WNV) is endemic to Africa, Asia, Europe, and Australia and was introduced into the Western Hemisphere in 1999. In the Northern Hemisphere, an apparent increase in human case fatality rates, neurologic infections, and horse and bird deaths due to WNV has raised the question whether WNV strains with increased pathogenicity have emerged in the Northern Hemisphere, or whether the virulence of the virus and the severity of the disease are underestimated in South Africa.

Two major phylogenetic lineages of WNV have been demonstrated: lineage 1 includes viruses from North Africa, Europe, Asia, the Americas, and Australia (Kunjin virus); lineage 2 consists exclusively of viruses from southern Africa and Madagascar. The increase in illness and death from WNV lineage 1 strains relative to lineage 2 strains led to the supposition that lineage 1 strains are highly pathogenic while lineage 2 strains endemic to Africa are of low virulence (*1**,**2*). However, it was subsequently demonstrated in South Africa that lineage 2 strains may also cause severe disease (*3*). Furthermore, experiments using mice demonstrated marked differences in neuroinvasive phenotype that did not correlate with lineage, which suggests that highly and less neuroinvasive phenotypes exist in both lineages (*2**,**4*). Host gene expression studies indicated that similar genes are induced by highly neuroinvasive lineage 1 and 2 strains (*4*). Therefore, the perceived virulence of WNV in recent epidemics probably reflects high medical alertness, active surveillance programs, and the emergence and reemergence of existing strains of WNV in locations with immunologically naive populations (*3*). Recently, a lineage 2 strain was isolated from a goshawk fledgling that died of encephalitis in Hungary, which suggests that lineage 2 strains may also be spread by migratory birds outside of Africa (*5*).

Lineage 1 viruses that have phenotypes of reduced virulence in mice and inefficient growth in culture have been identified in Mexico. Mutations leading to loss of envelope (E) protein glycosylation together with mutations in the nonstructural (NS) protein genes may be associated with attenuation of these viruses (*6*). Comparisons between the prototype Uganda strain (B956) and a variant of this strain, which was obtained by molecular mutation (B956D117B3), showed changes in the E and NS genes, which resulted in reduced virulence in mice (*7*). These attenuations could not, however, be correlated with clinical disease in humans because these strain were either isolated from birds or modified in culture.

The NS4B protein may play an important role in virulence phenotype determination (*6**,**8**–**10*), predicted to be involved in viral replication and evasion of host innate immune defenses (*8*). Substitution of cysteine at position 102 with serine (Cys102Ser) led to the formation of a temperature-sensitive phenotype at 41°C as well as attenuation of the neuroinvasive and neurovirulent phenotypes in mice (*8*). An adaptive mutation (E249G) in the NS4B gene resulted in reduced RNA synthesis in host cells (*9*). An in vitro study compared infectious clones of the NY99 strain, which is highly virulent in American crows, with a Kenya strain (KEN-3829), which is less virulent for American crows. After 72 days at 44°C, reduction in viral RNA production by the KEN-3829 strain was 6,500-fold, compared with the NY99 strain reduction of 17-fold. This finding suggested that efficient replication at high temperatures, as occurs in American crows, could be an important virulence factor that determines the pathogenic phenotype of the NY99 strain (*10*).

To further investigate the molecular determinants of virulence of lineage 2 WNV strains, we sequenced the genomes of highly and less neuroinvasive lineage 2 strains that were isolated from patients in South Africa and that had previously been characterized with respect to gene expression and pathogenicity (*4*). These complete genome sequences of highly neuroinvasive lineage 2 WNV strains enable comprehensive comparison with highly and less neuroinvasive lineage 1 strains.

## Materials and Methods

### Virus Strains

South African WNV isolates SPU116/89, SA93/01, SA381/00, and H442 were obtained from the Special Pathogens Unit, National Institute for Communicable Diseases, South Africa, as freeze-dried mouse brain passages 2–4. They were replicated by 1 passage in Vero cells for this study.

### RNA Amplification

Viral RNA was extracted from cell culture supernatant with the QIAamp Viral RNA Mini Kit (QIAGEN, Hilden, Germany) according to the manufacturer's instructions. For cDNA synthesis, 10 μL of RNA and 0.4 μg of random hexanucleotides (Roche Diagnostics, Mannheim, Germany) were incubated at 65°C for 10 min before cooling on ice. Then 1× Expand Reverse Transcriptase buffer, 100 mmol/L dithiothreitol, 200 μmol of each deoxynucleotide triphosphate, 20 U RNase Inhibitor and 50 U Expand Reverse Transcriptase (Roche Diagnostics) were added and incubated at 30°C for 10 min, followed by 1 h at 43°C. For PCR amplification, 10 μL of the cDNA reaction was added to the PCR master mix consisting of 3.75 U of Expand High Fidelity Polymerase and 30 pmol of each specific primer (primer sequences available on request) and cycled as follows: 94°C for 2 min (94°C for 15 s, followed by primer-specific annealing temperature for 30 s, 72°C for 2 min) × 35 and 72°C for 7 min. Expand Long Template PCR Polymerase (Roche Diagnostics) was used for products >2 kb with 300 μmol of each dNTP, 1× buffer, and 30 pmol of each specific primer and cycled at 94°C for 2 min (94°C for 10 s, 50°C for 30 s, 68°C for 3 min) × 10; followed by 30 cycles of 94°C for 15 s, 50°C for 30 s, 68°C for 5 min plus 5 s per cycle, and 72°C for 7 min.

### DNA Sequencing

PCR products were purified with Wizard SV gel and PCR clean-up system (Promega, Southampton, UK). DNA cycle sequencing was performed with the BigDye Terminator V3.1 kit and analyzed on an ABI PRISM 3100/3130 genetic analyzer (both from Applied Biosystems, Foster City, CA, USA).

### Sequence Analysis

Genome editing and assembling were performed by using Vector NTI 9.1.0 (Invitrogen, Carlsbad, CA, USA); multiple sequence alignments, with ClustalW (*11*); and amino acid analysis, with GeneDoc for Windows (*12*). Amino acid changes considered to have a potential effect on the secondary structure of the proteins included substitution of hydrophilic for hydrophobic amino acids or vice versa and substitutions of cysteine, glycine, and proline residues (*12*).

Comparisons are relative to the top sequence (SA381/00); numbering refers to the sequence position of isolate SA381/00. Neighbor-joining trees were drawn with MEGA version 3.1 (*13*) by using the Kimura-2 distance-parameter and a bootstrap confidence level of a 1,000 replicates. Nucleotide and amino acid p-distances (the number of pairwise nucleotide or amino acid differences divided by the total number of nucleotides or amino acids in the sequenced region) were calculated by using MEGA version 3.1. Signalase cleavage predicted scores were calculated with AnalyzeSignalase 2.03 (*14*).

## Results

### Strain Characteristics

Four lineage 2 WNV strains isolated from patients in South Africa who had mild or severe WNV infections were selected for genome sequencing. Phenotypic pathogenicity data for these strains (H442, SPU116/89, SA93/01, SA381/00) in humans and mice are summarized in Table 1. Detailed clinical data for all 4 strains have been described by Burt et al. (*3*), and mouse neuroinvasive experiments and gene expression data for H442, SPU 116/89, and SA381/00 have been described by Venter et al. (*4*). Strain SA93/01 has been shown to be highly neuroinvasive in a mouse model (M. Venter, unpub. data), similar to SPU116/89 and H442 strains, whereas SA381/00 has been classified as being of low neuroinvasive phenotype in mice. H442 and SA381/00 caused fever, rash, myalgia, and arthralgia in human patients; SA93/01 caused nonfatal encephalitis in 2, and SA116/89 caused fatal hepatitis (*3*).

**Table 1:** Characteristics and origin of West Nile virus strains included in this investigation*

Strain, year of isolation	Passage	Source	Location	Syndrome	Outcome	L	Neuro	Glyco	Ref	GenBank
SPU116/89, 1989	Mouse 3	Human	SA	Necrotic hepatitis	Died	2	High	NYS†	This study	EF429197
SA93/01, 2001	Mouse 1	Human	SA	Fever, rash, myalgia, encephalitis	Survived	2	High	NYS†	This study	EF429198
SA381/00, 2000	Mouse 1	Human	SA	Fever, rash, myalgia, arthralgia	Survived	2	Mild	NYS†	This study	EF429199
H442, 1958	Mouse 2	Human	SA	Fever, rash, myalgia, arthralgia	Survived	2	High	NYS†	This study	EF429200
B956, 1937	Mouse 2	Human	Uga	Febrile disease	Survived	2	Mild	Deletion of entire motif	(*7*)	AY532665
B956D117B3,‡ 1937	Unknown	Human	Uga	Febrile disease	Survived	2	Less than B956	Deletion of entire motif	(*15*)	M12294
Madagascar-AnMg798, 1978	Unknown	Parrot§	Mad	NA	Died	2	None	NYP	(*16*)	DQ176636
NY 385–99, 1999	Vero 2	Human	USA	Unknown	Unknown	1	High	NYS†	(*17*)	DQ211652
NY-385–99 Clone TYP-9376, 2005	Hamster	Hamster	USA	NA	NA	1	None¶	NYS†	(*18*)	AY848697
NY-385–99 Clone 9317B, 2005	Hamster	Hamster	USA	NA	NA	1	None¶	NYS†	(*18*)	DO66423
TM171–03, 2003	Vero 1	Common raven	Mex	Unknown	Died	1	None¶	NYP	(*19*)	AY660002
MRM61C, 1960	NA	Mosquito#	Aus	NA	NA	1	None	NYF	(*20*)	D00246

*L, lineage; Neuro, neuroinvasiveness in mice; Glyco, glycosylation of envelope protein; Ref., reference no.; GenBank, GenBank accession no.; SA, South Africa; Uga, Uganda; Mad, Madagascar; NA, not applicable; USA, United States; Mex, Mexico; Aus, Australia. †Strains that are glycosylated in the envelope protein positions 154–156. ‡A descendant of B956. §*Coracopsis vasa*. ¶Attenuated laboratory strains. #*Culex annulirostris*.

The 4 South African strains were compared with strains that were known to be highly or less neuroinvasive in mice or that had been reported to be highly pathogenic or attenuated. Lineage 2 strains for which both full genome sequences and neuronvirulence data in mice were available included isolate B956D117B3 (*21*) and Madagascar strain AnMg798. B956D117B3 is a passaged clone of reduced virulence (*7*) of the prototype strain (B956), which was originally associated with fever in a patient and was neurotropic in mice (*22*); AnMg798 is not neuroinvasive (*2*). Lineage 1 strains included the highly pathogenic and neuroinvasive NY385–99 strain (*2*), the attenuated non-neuroinvasive strain TM171–03 isolated in Mexico in 2003 (*19*), hamster-passaged attenuated clones of NY-385–99 (clone TYP-9376 and clone 9317B) (*18*), and a non-neuroinvasive Kunjin virus strain MRM61C (Table 1).

### Phylogenetic Analysis

**Figure 1:**
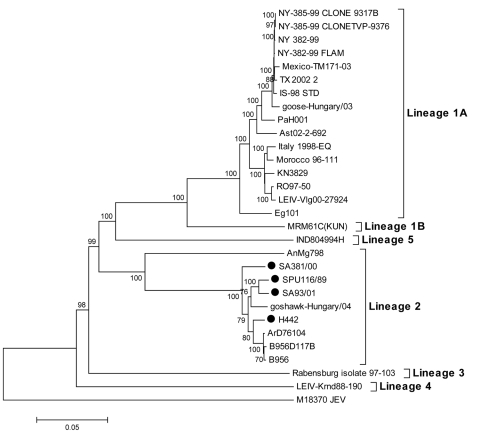
Phylogenetic analysis of full genome nucleotide sequences of lineage 1 and 2 strains of West Nile virus. The tree was constructed by using MEGA version 3.1 (*13*) with the neighbor-joining method and Kimura 2-parameter distance matrix. A bootstrap confidence level of 1,000 replicates was used. South African strains sequenced in this study are indicated by a black circle. GenBank accession nos. are as follows: NY-385–99 clone 9317B (DO66423), NY-385–99 clone TVP-9376 (AY848697), NY 385–99 (DQ211652), NY-382–99 FLAM (AF196835), IS-98 STD (AF481864), Mexico-TM171–03 (AY660002), TX 2002 2, (DQ 164205), goose-Hungary/03 (DQ 118127), Eg 101 (AF 260968), RO97–50 (AF260969), Morocco 96–111 (AY701412), Italy 1998-Eq (AF 404757), KN3829 (AY262283), LEIV-Vlg00–27924 (AY278442), PaH001 (AY268133), Ast02–2-696 (DQ411035), MRM61C (KUN) (D00246), IND804994H (DQ 256376), AnMg798 (DQ 176636), SA381/00 (EF429199), H442 (EF429200), goshawk-Hungary/04 (DQ 116961), SPU116/89 (EF429197), SA93/01 (EF429198), B956D117B3 (M12294), B956 (AY532665), ArD76104 (DQ 318019), Rabensburg isolate 97–103 (AY765264), LEIV-Krnd88–190 (AY277251), and M18370 JEV (M18370).

Phylogenetic analysis confirmed that the South African strains described here belong within lineage 2 (Figure 1). SA93/01 and SPU116/89 clustered together; H442 and SA381/00 were on separate branches within lineage 2 with respect to the full genome sequences or with respect to individual E, NS3, and NS5 genes (data not shown). Although the Indian strain clustered with lineage 1, p-distance analysis suggested that it was as distant to the lineage 1 strains (20% differences) as to the lineage 2 strains (21%–22%) relative to <5% differences within lineage 1C and 12% differences between 1A and 1B. It was therefore termed lineage 5, as suggested by Bondre et al. (*23*).

### Genome Sequences and Distance Analysis

The complete genome sequences of strains H442, SPU116/89, SA381/00, and SA93/01 were deposited in GenBank (accession nos. EF429197–200). The termini were amplified with primers designed from other lineage 2 full genome sequences. If one assumes that the 5′ and 3′ termini are identical in length to other published strains, these genomes were 11052 nt (SPU116/89, SA381/00, and SA93/01) and 11051 nt (H442) long. South African strains had overall nucleotide p-distances of 0.0278 (97.2% similarity) to each other (Table 2), with <1% amino acid differences over the complete genome despite having been isolated as many as 50 years apart. The highest percentage of amino acid differences in the individual proteins of the lineage 2 strains were in the NS proteins, especially the NS5 protein. Table 3 shows the differences between individual proteins of the South African lineage 2 strains.

**Table 2:** Percentage of amino acid and nucleotide differences when comparing the entire genome of selected West Nile virus strains*

Strain	SA381/00	H442	SPU116/89	SA93/01	B956D117B3	B956	ANMg798	NY-385–99	NY-385–99 Clone TYP-9376	NY-385–99 Clone 9317B	TM171–03	MRM61C
SA381/00		2.4	3.6	3.7	3.1	2.9	15.8	20.6	20.6	20.6	20.6	20.5
H442	0.7		2.7	3.0	1.8	1.6	15.6	20.5	20.6	20.6	20.5	20.6
SPU116/89	0.9	0.7		1.3	2.0	1.9	16.0	20.8	20.9	20.9	20.8	20.7
SA93/01	0.8	0.7	0.6		2.3	2.2	15.7	20.8	20.9	20.9	20.8	20.6
B956D117B3	1.0	0.8	1.0	0.9		0.3	15.7	20.7	20.7	20.7	20.7	20.6
B956	0.7	0.6	0.7	0.7	0.7		15.8	20.7	20.7	20.7	20.7	20.6
AnMg798	3.4	3.3	3.5	3.4	3.5	3.4		21.5	21.5	21.5	21.4	21.2
NY-385–99	6.0	5.9	6.1	6.1	6.1	6	6.6		0.1	0.1	0.4	11.7
NY-385–99 Clone TYP-9376	6.1	6.0	6.2	6.2	6.2	6.1	6.7	0.1		0	0.5	11.8
NY-385–99 Clone 9317B	6.1	6.0	6.2	6.2	6.2	6.1	6.7	0.1	0.0		0.5	11.8
TM171–03	6.0	5.9	6.1	6.1	6.1	6	6.7	0.1	0.2	0.2		11.8
MRM61C	6.5	6.5	6.7	6.6	6.6	6.5	7.1	2.4	2.4	2.4	2.4	

*The lower left matrix corresponds to amino acid sequences, and the upper right matrix corresponds to nucleotide sequences.

**Table 3:** Percentage amino acid and nucleotide differences when comparing individual proteins of selected West Nile virus strains*

Strain	SA381/00	H442	SPU116/89	SA93/01
Capsid				
SA381/00				
H442	0			
SPU116/89	0	0		
SA93/01	0	0	0	
Envelope				
SA381/00				
H442	0.8			
SPU116/89	0.2	0.6		
SA93/01	0.2	0.6	0	
NS2A/B				
SA381/00				
H442	0.6			
SPU116/89	0.3	0.8		
SA93/01	0.3	0.8	0	
NS4A/B				
SA381/00				
H442	0.2			
SPU116/89	0.2	0.5		
SA93/01	0	0.2	0.2	
prM				
SA381/00				
H442	0.6			
SPU116/89	0.6	0		
SA93/01	0.6	0	0	
NS1				
SA381/00				
H442	0.9			
SPU116/89	0.9	0		
SA93/01	1.1	0.3	0.3	
NS3				
SA381/00				
H442	0.8			
SPU116/89	0.6	0.5		
SA93/01	0.6	0.5	0	
NS5				
SA381/00				
H442	0.8			
SPU116/89	2.1	1.5		
SA93/01	1.9	1.3	2.0	

*prM, premembrane; NS, nonstructural.

The 2 strains from North America, New York (NY-385–99) and Mexico (TM171–03), were similarly conserved. In contrast, the Madagascar strain, AnMg798, differed by >3% at the amino acid level from all lineage 2 strains from South Africa or Uganda B956D117B3, and the lineage 1 and 2 strains differed by >6% amino acids from each other.

### Amino Acid Differences between Highly and Less Neuroinvasive Strains

**Figure 2:**
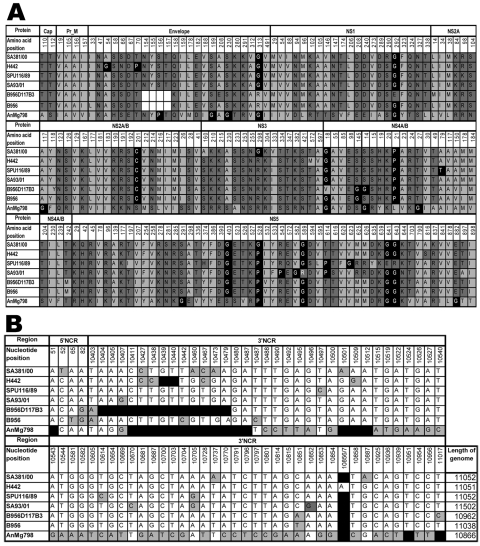
A) Amino acid differences between South African lineage 2 strains of West Nile virus (WNV) strains sequenced in the present study and previously published lineage 2 strains. Strain SA381/00 is less neuroinvasive than the highly neuroinvasive strains SA93/01, H442, and SPU116/89. Light gray, hydrophobic amino acids; dark gray, hydrophilic amino acids; black, structural-determining amino acids; white blocks, deletion of the glycosylation site in the envelope protein of the B956D117B3 strain. Numbering is according to the SA381/00 genome position for specific genes. Cap, capsid; prM, premembrane; NS, nonstructural. B) Nucleotide differences in the noncoding 5′ and 3′ regions of lineage 2 strains. Numbering is according to WNV strain SA381/00. Black, deletions; gray, nucleotide differences. The length of each genome is given in the last column. (Strain AnMg798 is incomplete in the GenBank database and may thus be longer than indicated.)

Few amino acid differences were observed between the structural proteins of the South African strains (Figure 2). SA381/00 had only 1 difference in the premembrane (prM) protein at position 105 relative to the highly neuroinvasive strains (Ala105Val) (Figure 2). Two differences, (Ala54Gly and Thr70Pro) could result in structural changes in the E protein of H442, which was isolated 50 years earlier than strains SPU116/89, SA93/01, and SA381/00. The attenuated lineage 2 strain B956D117B3 and the non-neuroinvasive Madagascar strain AnMg798 contained differences in the glycosylation site of the E protein relative to the South African strains (residues 154–157 deleted in B956D117B3, and Ser156Pro in AnMg798). Either of these changes would prevent glycosylation. Further substitutions of hydrophilic amino acids for proline and glycine residues with potentially structural implications were found in AnMg798 at positions 156, 199, and 230.

The NS3, NS4A/B, and NS5 proteins were the most variable viral proteins. In strain SA381/00, the least virulent of the 4 strains, a hydrophobic amino acid in contrast to a hydrophilic amino acid (Ser160Ala) and Arg298Gly could alter the structure of the SA381/00 NS3 protein. In the highly pathogenic strain SPU116/89, a hydrophobic-to-hydrophilic mutation (Ala79Thr) in NS4B is found relative to the other strains. Other amino acid changes with potential structural implications were for strain B956D117B3 at positions 18 and 145 of the NS4A gene and 14 of the NS4B gene and for strain AnMg798 at positions 14 and 27 in the NS4B gene of (Figure 2).

The NS5 protein was the most variable. Several positions were identified where the South African strains associated with mild infections (SA381/00 and H442) and the 2 other lineage 2 strains (AnMg798,B956D117B3) associated with reduced virulence in mice had the same amino acid changes relative to strains that caused severe disease (SPU116/89 and SPU93/01). These included hydrophilic versus hydrophobic amino acids in position 614 and hydrophobic (mild) versus hydrophilic (pathogenic) in positions 625 and 626 of the NS5 protein. SPU116/89, isolated from a patient with necrotic hepatitis, was found to have amino acid changes that affect the hydrophobicity of the NS5 protein relative to all other strains in positions 197, 623, 635, 641, and 643.

### Noncoding Regions

Approximately 98.6% identity existed between the 5′ noncoding regions of SA381/00 and the 3 remaining South African strains; the other strains were 100% conserved. For the 3′ noncoding regions, the overall identity was 98.5% (99% between SPU381/00 and H442 and 98% between SPU116/89 and SA381/00). Noteworthy nucleotide differences in the 3′ noncoding regions were a 2-nt deletion at nt 10439 and nt 10440 in strain H442 and a 76-bp deletion in the 3′ noncoding region from nt 10404 through nt 10479 in the attenuated strain B956D117B3, which was not present in the prototype strain (B956) or in any of the South African strains. Strain AnMg798 had deletions overlapping those of strain B956D117B3 at position 10411 to 10487 and from 10501 to 10512 and 10951 (Figure 2, panel **B**). The sequence of the AnMg798 strain is incomplete in GenBank and ended at position 10866 (*16*).

### Envelope-Protein Glycosylation Motif

The E protein glycosylation motif previously identified in lineage 1 at positions 154–156 (NYS) (*6*) was present in all 4 South African strains. However, as a result of a proline substitution at position 156, the site was not predicted to be glycosylated in strain AnMg798. The glycosylation motif is deleted completely in strains B956 and B956D117B3 (Table 1).

### Cleavage Sites

Signalase prediction algorithms were used to analyze the signal peptidase cleavage sites (*14*); no differences were found in cleavage efficiency between the highly and less pathogenic strains (Table 4). The only meaningful difference was observed in the capsid (C)–PrM cleavage region, as indicated by the Student *t* test probability calculated in Table 4, where the lineage 2 strains were predicted to be cleaved more efficiently than lineage 1 stains. Only slight differences were apparent in the PrM-E site; no differences were apparent in any other cleavage regions between lineage 1 and 2 strains.

**Table 4:** Summary of cleavage scores predicted for cleavage junctions of proteins of West Nile virus strains*

	SA381/ 00	H442	SPU116/ 89	SA93/01	B956D117B3	AnMg798	NY-385–99	NY-385–99 clone TYP-9376	NY-385–99 clone 9317B	TM171–03	MRM61C	p value†
Between capsid and premembrane proteins												
G	+3.69	+3.69	+3.69	+3.69	+3.69	+4.00	−0.49	−0.49	−0.49	−0.49	−1.85	0.00005
A‡	+9.37	+9.37	+9.37	+9.37	+9.37	+8.01	+5.93	+5.93	+5.93	+5.93	+7.37	0.00004
V	−9.14	−9.14	−9.14	−9.14	−9.14	−7.8	−9.52	−9.52	−9.52	−9.52	−10.32	0.02235
Between premembrane and envelope proteins												
Y	−10.15	−10.15	−10.15	−10.15	−10.15	−9.12	−9.12	−9.12	−9.12	−9.12	−9.45	0.00433
S‡	+11.27	+11.27	+11.27	+11.27	+11.27	+12.42	+12.42	+12.42	+12.42	+12.42	+11.50	0.01728
F	−5.37	−5.37	−5.37	−5.37	−5.37	−4.78	−4.78	−4.78	−4.78	−4.78	−5.27	0.01977
Between envelope protein and nonstructural protein 1												
H	−9.01	−9.01	−9.01	−9.01	−9.01	−9.71	−9.01	−9.01	−9.01	−9.01	−9.01	0.36322
A‡	+4.26	+4.26	+4.26	+4.26	+4.26	+4.04	+4.26	+4.26	+4.26	+4.26	+4.26	0.36322
D	−11.05	−11.05	−11.05	−11.05	−11.05	−11.15	−11.05	−11.05	−11.05	−11.05	−11.05	0.36322
Between nonstructural proteins 4B and 5												
R	−16.05	−16.05	−16.05	−16.05	−16.05	−16.05	−16.05	−16.05	−16.05	−15.83	−16.05	0.37390
G‡	−13.19	−13.19	−13.19	−13.19	−13.19	−13.19	−13.19	−13.19	−13.19	−13.08	−13.19	0.37390
G	−19.54	−19.54	−19.54	−19.54	−19.54	−19.54	−19.54	−19.54	−19.54	−19.66	−19.54	0.37390

*Signal cleavage predicted scores were calculated with AnalyzeSignalase 2.03 (*14*). The table indicates only the last amino acid of the first protein and the first 2 amino acids of the following protein. †Two-tailed Student *t* test results, indicating the probability of significance of observed differences between lineage 2 and lineage 1 strains. ‡Exact cleavage site.

## Discussion

Phylogenetic and p-distance analyses suggested that relationships between WNV strains were influenced by geographic rather than temporal factors (Figure 1, Tables 2, 3). Four South African strains isolated over 50 years differed from each other by an average of only 3% of nucleotides but from the AnMg798 (Madagascar) strain by 21%.

The WNV genome consists of a 5′ noncoding region, a single open reading frame coding for 3 viral structural proteins (C, M, and E) and 7 NS proteins, and a 3′ noncoding region. The E and membrane (M) proteins are associated with host range, tissue tropism, replication, assembly, and the stimulation of the B- and T-cell immune responses; replication functions are associated with the NS proteins, which may also modulate responses to viral infection (*6*). The E protein is the viral hemagglutinin that mediates virus–host cell binding and elicits most of the virus neutralizing antibodies and serotype specificity of the virus (*1**,**24**,**25*).

In this study, differences between highly and less neuroinvasive lineage 2 stains were identified in the noncoding regions, which may potentially affect enzyme binding sites and replication efficiency (Figure 2, panel **B**). It has been postulated that the 3′ stem loop structure may function as a translation suppressor (*26*) and that nucleotide sequence variation in the 3′ noncoding region of different dengue strains may have evolved as a function of transmission or replication ability in different mosquito and nonhuman primate/human host cycles (*27*). A 76-bp deletion in the 3′ noncoding region is present in strains B956D117B3 and AnMg798 relative to the South African strains. This deletion is not present in the original neurotropic mouse brain isolate of the B956 Uganda strain, which has recently been resequenced (*7*). Strain B956D117B3, a descendent of the original B952 isolate, has been shown to be less virulent than the original B956 strain. The absence of this deletion in all of the neuroinvasive lineage 2 strains isolated from clinical cases warrants further investigation of the role of the region in the pathogenicicty of WNV.

The genetic stability observed in the surface E and M proteins of lineage 2 strains suggests an absence of immune-driven selection. Only the H442 strain, isolated 50 years before the other strains, had 2 substitutions in the E gene with potential structural implications (Figure 2). The absence of a putative E protein glycosylation site at positions 154–156 of the E protein (NYS) has previously been associated with reduced virulence in mice (*19*). This glycosylation motif was present in all the South African strains, including the less neuroinvasive strain SA381/00. However, the prototype lineage 2 strain B956D117B3 and the non-neuroinvasive lineage 1 and 2 strains MRM61C and AnMg798 were not glycosylated. This finding further emphasizes that glycosylation of the E protein is not the only determining factor for virulence.

Most substitutions were found in the NS proteins, in particular NS3, NS4A/B, and NS5. The NS3 protein is part of the protease complex, which is important for cleavage of the polyprotein and may affect virulence; it has been suggested that less efficient cleavage results in delayed virus assembly and release, enabling the host immune system to clear infection (*28*). The NS3 protein of the less neuroinvasive strain, SA381/00, manifested hydrophobic and hydrophilic changes, which could lead to structural changes that affect function and, by implication, virulence. The highly neuroinvasive strain SPU116/89 had mutations that may alter the hydrophobicity of the NS4B protein (Ala79Thr) relative to the other strains and may have potential structural and functional implications for the viral replicase complex of which NS4B is a component (*25*).

Most amino acid differences occurred in the NS5 protein, which is associated with cytoplasmic RNA replication because it contains an RNA-dependent RNA polymerase, *S*-adenosylmethionine methyltransferase, and importin β–binding motifs (*28*). Deletions in the NS5 protein abolish replication (*29*), which suggests that amino acid substitutions may effect replication efficiency and, hence, virulence. Temperature-sensitive strains with reduced virulence for mice, isolated in Texas, also contained mutations in the NS proteins (*30*). In addition, organ tropism of strains has been associated with mutations in the NS5, NS2, and E proteins (*18*). The 2 lineage 2 strains that caused mild disease in patients (H442 and SA381/00) had several substitutions of hydrophobic to hydrophilic amino acids relative to the other 2 strains in the NS5 protein. SPU 116/89, isolated from a patient with necrotic hepatitis, had several amino acid changes that may affect its hydrophobicity and result in structural and functional changes that have implications for altered replication efficiency, tissue tropism, and pathogenicity.

Flavivirus polyproteins are cleaved either by a host signal peptidase or a viral-encoded serine protease consisting of the NS3 protease and the NS2B cofactor (NS2B-NS3) (*29*). Proteolytic processing of the C-prM and NS4A/B proteins occurs efficiently only after upstream cleavage of the signal sequence by cytoplasmic viral protease. Efficiency of signal peptidase cleavage at the NH_2_ termini of prM and NS4 proteins is increased by coexpression of the viral NS2B-NS3 protease and the structural polyprotein region (*31*). Mutagenesis analysis of the signal sequence of yellow fever virus prM protein indicated that mutations that enhance cleavage by the signal peptidase almost totally suppress production of infectious virions (*31*). Signal peptidase cleavage of prM protein results in the production of membrane-anchored forms of the C protein, which may be deleterious for replication if it functions poorly as a substrate for viral protease. The signal peptidase-mediated cleavage at the NH_2_ terminus of prM protein does not occur efficiently, whereas cleavage at the NH_2_ terminus of the E protein does. Inadequate prM protein production in turn affects production and lowers the secretion of prM-E heterodimers. When these constructs are used in vaccination studies, a lack of immunogenicity is noted (*32*).

In the present study, all highly and less pathogenic lineage 2 strains as well as lineage 1 strains were predicted to be cleaved with the same efficiency. At the C-prM site, lineage 2 strains are cleaved slightly more efficiently than lineage 1 strains; at prM-E, the reverse is true. How these differences in cleavage efficiency affect pathogenicity is unclear and may warrant further investigation.

The high number of cases of neurologic infections in recent epidemics in the United States may be attributed to the rapid distribution of a single highly neuroinvasive strain in a highly susceptible population. The comparatively low number of WNV fever or neurologic cases reported in South Africa, despite the wide distribution of the virus and the presence of neuroinvasive strains, may reflect inadequate surveillance and a lack of medical awareness of the disease potential of arboviruses. Moreover, the importance of WNV in South Africa may be overshadowed by the presence and effect of other diseases such as HIV/AIDS. Nevertheless, the epidemic potential and effect that WNV may have on a large population of immunocompromised HIV-infected persons necessitates improved surveillance of arbovirus infections of persons in southern Africa.

In conclusion, these full genome sequences provide insight into the molecular factors that may differentiate pathogenic from mild lineage 2 WNV strains. Mutations in the NS proteins encoding viral replication and protein cleavage mechanisms are the most likely determinants of differences in pathogenicity.
